# Maglev Train Signal Processing Architecture Based on Nonlinear Discrete Tracking Differentiator

**DOI:** 10.3390/s18061697

**Published:** 2018-05-24

**Authors:** Zhiqiang Wang, Xiaolong Li, Yunde Xie, Zhiqiang Long

**Affiliations:** 1 Maglev Engineering Research Center, National University of Defense Technology, Changsha 410073, China; wangzhiqiang12@nudt.edu.cn (Z.W.); 13787786254@163.com (X.L.); 2 Beijing Enterprises Holdings Maglev Technology Development Co. Ltd., Beijing 100124, China; xieyd9999@163.com

**Keywords:** maglev train, signal processing architecture, tracking differentiator (TD), FPGA

## Abstract

In a maglev train levitation system, signal processing plays an important role for the reason that some sensor signals are prone to be corrupted by noise due to the harsh installation and operation environment of sensors and some signals cannot be acquired directly via sensors. Based on these concerns, an architecture based on a new type of nonlinear second-order discrete tracking differentiator is proposed. The function of this signal processing architecture includes filtering signal noise and acquiring needed signals for levitation purposes. The proposed tracking differentiator possesses the advantages of quick convergence, no fluttering, and simple calculation. Tracking differentiator’s frequency characteristics at different parameter values are studied in this paper. The performance of this new type of tracking differentiator is tested in a MATLAB simulation and this tracking-differentiator is implemented in Very-High-Speed Integrated Circuit Hardware Description Language (VHDL). In the end, experiments are conducted separately on a test board and a maglev train model. Simulation and experiment results show that the performance of this novel signal processing architecture can fulfill the real system requirement.

## 1. Introduction

**Figure 1:**
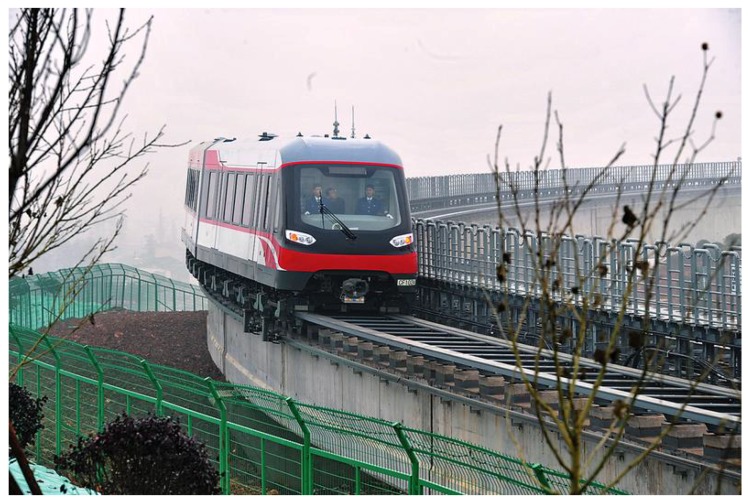
Changsha Maglev Express train in a small-radius circle.

The magnetic levitation (maglev) train is a kind of promising transportation system that replaces wheels by electromagnets and levitates on the guideway via non-contact electromagnetic force. It offers numerous advantages over the conventional wheel-on-rail system: (1) it is suitable to operate as a city transportation tool without disturbing the residents due to its low noise; (2) electromagnets and specially designed tracks prevent the trains from derailing; (3) its property of small turning radius and strong climbing ability lowers the difficulty for route planning in mountainous landscapes and densely populated areas; and (4) it provides a consequent reduction in maintenance costs, and so forth [1,2]. Recent running maglev trains in the Changsha Maglev Express (Figure 1) and the Beijing Maglev S1 have shown its widespread application prospects.

In a maglev levitation system, a dynamic regulated electromagnet force is utilized to neutralize the load. To accomplish this control purpose, it is necessary to acquire sufficient information about the levitation system as the feedback, so as to calculate the control output based on all this feedback. The system information necessary for control purposes is usually acquired directly or indirectly from sensors in the maglev train. Currently, the original sensor signals available contain the levitation gap of the train from the gap sensor, vertical acceleration of the electromagnet from the accelerator, and the current in the electromagnet windings from the current sensor. The quality of the system information plays an important role in improving levitation performance since the information is served as feedback for control purposes. Therefore, a signal processing unit that is in charge of offering sufficient information for control output computation based on the original sensor signals is indispensable. The main challenges for this signal processing unit can be categorized as follows:

(1) Since there exist inevitable electromagnetic disturbances, which exert an influence on the working condition of the gap sensor, the gap sensor generates not only real signals that reflect the real gap information, but also noise that may influence the normal functioning of the controller if this noised gap sensor is utilized directly as the feedback without preprocessing. To minimize the influence caused by this kind of noise disturbance, a gap sensor signal must be filtered before it is used by the controller. At present, most commonly used filters for gap signals are simple first-order low-pass filters, for which the filtering performance and the real-time requirement contradict each other. To enhance the filter’s performance, a big filtering coefficient is required, whereas a big filtering coefficient usually causes a large phase lag that undermines the stabilities of the levitation system [3]. A new filtering strategy with higher filtering performance and smaller phase lag is needed.
(2) Train-track coupled vibration is a special phenomenon for a maglev system due to the elastic property of the steel track [4]. It is found that if the information of the movement of the track is obtained for computation of the control output, this vibration can be effectively suppressed [5,6]. Since the differential of gap is the relative velocity between the electromagnet and the track, this information is effective for suppression of this kind of vibration. However, the acquisition of differential for a discrete signal is a difficult task. The differential of a given discrete signal can amplify the noise, especially the high frequency noise. Sometimes, the real differential result can be overwhelmed by this amplified noise. Therefore, a differentiator that can acquire the differential for signals within the given frequency region without amplifying the high frequency noise is needed.
(3) Model based signal processing strategy is effective in filtering and obtaining derivative signals under the condition that the object model is time invariant and precisely known a priori; however, the maglev model is sometimes not precise and is always time varying due to the changing passenger amount and the varying relative position between the electromagnet and the track [7]. For this reason, the signal processing method with less dependence on the system model is more suitable for the maglev train levitation system.

Based on the particular requirement of the maglev train levitation system on signal processing tasks, the tracking-differentiator (TD) with the property of fast tracking and a small phase lag is an appropriate choice. Filtering and the differential of the input signal can be accomplished at the same time via a tracking differentiator. The tracking differentiator began to attract researchers’ attention in the late 1980s due to pursuing the high performance of control systems [8,9]. Since then, much effort has been devoted to the problem of designing a differentiator, such as a high-gain observer-based differentiator, linear time-derivative tracker, super-twisting second-order sliding-mode algorithm, robust exact differentiator, and finite time-convergent differentiator among others [10]. Levant proposed differentiators based on slide-mode techniques, which highlighted the later development of tracking differentiator [11,12]. In this kind of differentiator, the upper bound for Lipschitz constant is needed. However, the output of derivative estimation is not smooth because of the existence of discontinuous function. Therefore, the chattering phenomenon exists in the derivative estimation. In some works, the global robust exact differentiator was designed by combining the high-gain differentiator with sliding modes differentiation though a switch function [13,14]. Wang Xinhua proposed a hybrid continuous nonlinear differentiator in which the chattering phenomenon can be reduced sufficiently [15]. Angulo et al. proposed a tracking differentiator that could uniformly converge with initial differentiator error and finite-time exact convergence [16]. Utkin has also done a series of studies on the features of sliding differentiator [17,18].

Since the digital controller is widely used in the maglev train levitation control system as well as in other industry control systems, the research on the discrete-form tracking differentiator is of more practical importance. Han put forward a noise-tolerant time-optimal system based practical discrete tracking differentiator in early time [19]. The advantage of this TD is that it sets a weak condition on the stability of the systems to be constructed for TD and requires a weak condition on the input. In addition, it has advantageous smoothness compared with the obvious chattering problem encountered by sliding-mode-based differentiators [20]. Yao Guo uses a tracking differentiator for compass signal tracking [21]. Dai and Xue apply tracking differentiators in the field of filtering for sensor signals [22,23]. Zhang makes use of the tracking differentiator in the field of X-ray pulsar profile recovery [24]. Zhang et al. make use of the tracking differentiator to get a transient profile, improving power control performance of wind generation system [25]. Dou et al. solve a problem of filtering and direction identification in relative position estimation based on induction loop-cable using a tracking differentiator [26]. These works demonstrate the widespread interest in tracking differentiators. Further research about tracking differentiators is of great importance in promoting its practical application.

A few new forms of tracking differentiators are also proposed in recent research [27,28,29,30,31]. However, many works mentioned above present only simulation results and the forms of tracking differentiators in these papers are much too complicated. In addition, in the maglev train levitation control system, an Field-Programmable Gate Array (FPGA) processor is responsible for signal processing, and no literature has given a method to implement a tracking differentiator in an FPGA processor.

This article gives a further study of tracking differentiator, which is concise in its form and easy to be implemented in hardware. A maglev train signal processing architecture based on this novel tracking differentiator is described in detail. Influences of different parameters on the tracking differentiator’s performance and effect of this signal processing architecture on the maglev train levitation system are analyzed then via MATLAB simulations (MATLAB 8.3, MathWorks, Natick, MA, USA). Following this, a Very-High-Speed Integrated Circuit Hardware Description Language (VHDL) implementation of this tracking differentiator is made and a MATLAB-Modelsim simulation is conducted to verify the VHDL implementation’s effectiveness. In the end, a maglev test bench is used to verify the effectiveness of the tracking differentiator based signal processing architecture.

## 2. Signal Processing Architecture Based on Tracking Differentiator

**Figure 2:**
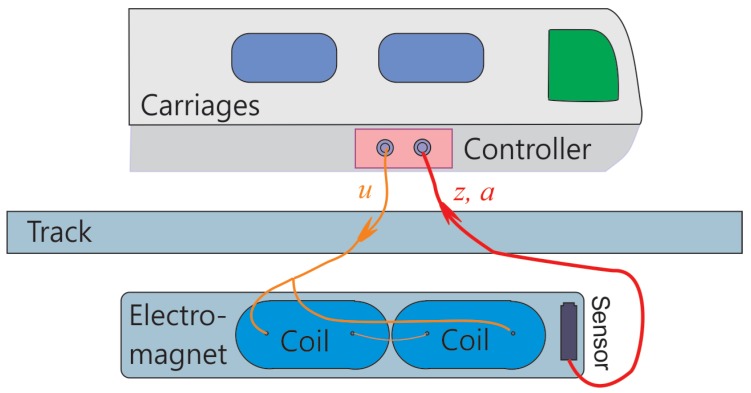
Signal flow chart in a maglev train levitation system.

A simplified maglev system is presented in Figure 2, which consists of carriages, track, electromagnet and sensor. In this simplified maglev system, the sensor embedded on the electromagnet is an integrated sensor offering gap signal and acceleration signal. It acquires gap between the lower surface of the track and the upper surface of the electromagnet, the acceleration of the electromagnet and transmits these signals to the controller via field bus. The current sensor and the voltage sensor are located inside the controller, which offer the current value through the coil and the voltage exerted on the coil. After acquiring these sensor signals, the controller computes control output based on particular control law and then generates output voltage onto the coil. When there is voltage exerted, current is generated inside the coil, and the magnetic force levitates the electromagnet upwards together with the carriages. During this process, the controller regulates the output voltage dynamically to maintain the balance between the electromagnetic levitation force and the weight of the carriage.

Under ideal condition, assuming:

- there is no magnetic flux leakage,
- there is no external disturbance force,
- there is only the vertical movement of the electromagnet,
- the track is rigid,

a linearized model of the maglev system can be derived as [2]:

(1) $$\left[\begin{array}{c} \dot{z}\\ \dot{v}\\ \dot{i} \end{array}\right]=\left[\begin{array}{ccc} 0 & 1 & 0\\ \frac{k_{z}}{m} & 0 & \frac{k_{c}}{m}\\ 0 & 0 & -\frac{R}{L_{0}} \end{array}\right]\left[\begin{array}{c} z\\ v\\ i \end{array}\right]+\left[\begin{array}{c} 0\\ 0\\ \frac{1}{L_{0}} \end{array}\right]u_{c},$$

where z,v, and *i* are the system state variables representing displacement of the electromagnet, velocity of the electromagnet, and current inside the windings , $u_{c}$ is the control voltage, *m* and *R* represent the mass of the electromagnet and resistance of the windings, $k_{z}$ , $k_{c}$ and $L_{0}$ are the gap coefficient, current coefficient and inductance of the electromagnet windings at the equilibrium point. Ideally, a state feedback type control law (2), which is a linear combination of system states with $K_{z},K_{v},$ and $K_{c}$ denoting the displacement coefficient, velocity coefficient, and current coefficient, respectively, can stabliize the maglev system described in Equation (1):

(2) $$u_{c}=K_{z}z+K_{v}v+K_{c}i.$$

In the real system, the displacement *z* is substituted by the levitation gap δ from the gap sensor, and the velocity *v* is substituted by the integral of the acceleration *a* from the accelerator. Additionally, to reduce static error, the integral of the levitation gap error is also included into the control output. Thus, Equation (2) can be transformed into:

(3) $$u_{c}=K_{z}\delta +K_{v}\int a+K_{c}i+K_{i}\int \left(\delta -z_{ref}\right),$$

where $z_{ref}$ is the target levitation gap, and $K_{i}$ is the coefficient of the integral term. In this control strategy, the quality of the sensor signal plays an important role, especially the quality of levitation gap signal and its differential. For current signal, (1) current sensor is installed in the controller on the train body instead of the electromagnet, the electromagnetic disturbance for current sensor is weak compared with that for gap sensor and accelerometer; (2) the coefficient for gap and velocity is far larger than the coefficient for current, which means the influence of noise in the current sensor is not comparable with that of noise in gap sensor. Supposing the original current signal time series are $i_{0}\left(k\right),k=1,2,3,\cdot \cdot \cdot$, then a two-step current signal processing method represented by Equations (4) and (5) is adopted, with $\tilde{i}\left(k\right)$ the filtered result:

(4) $$\begin{array}{c} i_{1}\left(k\right)=i_{0}\left(k\right)+i_{0}\left(k-1\right)+i_{0}\left(k-2\right)-\max\left(i_{0}\left(k\right),i_{0}\left(k-1\right),i_{0}\left(k-2\right)\right)\\ \;\;\;\;\;\;\;\;-\min(i_{0}\left(k\right),i_{0}\left(k-1\right),i_{0}\left(k-2\right))\;k=3,4,5,\cdot \cdot \cdot , \end{array}$$

(5) $$\tilde{i}\left(k\right)=\left\{\begin{array}{c} i_{1}\left(k-1\right)\;if\left|i_{1}\left(k-1\right)-i_{1}\left(k\right)\right|>1,\\ i_{1}\left(k\right)\;if\left|i_{1}\left(k-1\right)-i_{1}\left(k\right)\right|\leq 1. \end{array}\right.\;k=4,5,6,\cdot \cdot \cdot ,$$

For the acceleration signal, it is integrated as velocity feedback. Since noise is usually treated as an additive white noise, and the integral of white is close to zero, the influence of noise in the acceleration signal is trivial. Sensor biases, which may become a big problem if the biased acceleration signal is processed by the integral directly, should be filtered first. Considering the fact that acceleration of the electromagnet is supposed to be of high frequency, a first order high pass filter (6) is used for processing of the acceleration signal, which is also effective in filtering low frequency sensor biases:

(6) $$G_{hp}\left(s\right)=\frac{s}{s+T_{a}},$$

where $T_{a}$ is a constant related with the threshold of the high pass filter. Both the processing of current signal and acceleration signal is easy, the challenge is on the processing of the gap signal. The eddy current type gap sensor is located next to the electromagnet windings, and the magnetic field and the heat generated by the electromagnet windings cause disturbances to the acquisition of the gap signal and the acquisition of its differential. To get rid of the influence of sensor noise, the first order low-pass filter (7) is commonly used, in which $T_{f}$ is the time constant. The disadvantage is that, when a high filtering quality is needed, the phase lag is relatively too large, which has a bad influence on system stability. The task is to find a filter with a good filtering performance and small phase lag as well:

(7) $$G_{f}\left(s\right)=\frac{1}{T_{f}s+1}.$$

For acquisition of the gap differential signal, a classic method (8) is commonly used. In which the smaller $T_{d}$ is, the closer is (8) to the real differential value of the given signal. However, if there exist noise in the given signal, then there will be $1/T_{d}$ times this noise in the differentiated result. Since $T_{d}$ is usually pretty small, this noise will be amplified dramatically. So the task is to find a differentiator which will not amplify high frequency noise:

(8) $$G_{d}\left(s\right)=\frac{1}{T_{d}}\left(1-\frac{1}{T_{d}s+1}\right).$$

**Figure 3:**
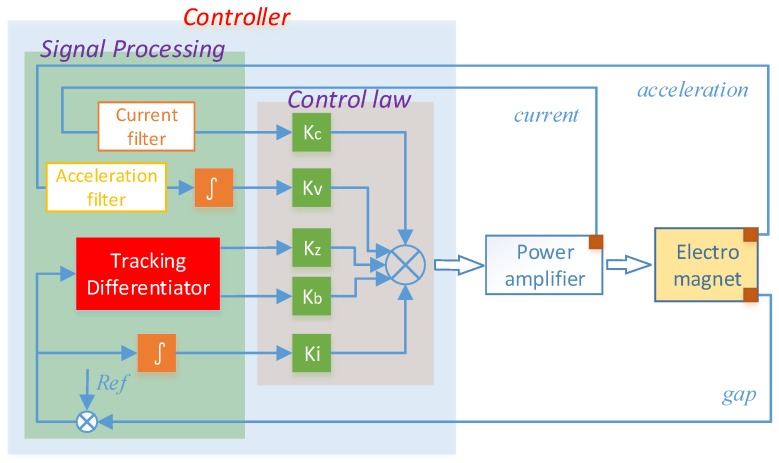
Maglev train signal processing architecture based on the tracking differentiator.

Based on the analysis above, a maglev train signal processing architecture based on the tracking differentiator is proposed (Figure 3).

The levitation gap sensor and accelerator, which are embedded on the electromagnet transmit levitation gap signal and acceleration signal to the controller, the current sensor transmits current signal to the controller. These are all the original signals to be processed. In the controller, these signals are processed for computation of control output. The acceleration signal *a* is processed first by acceleration filter (6), and then the filtered signal $\tilde{a}$ is integrated as the absolute velocity feedback; the current signal is processed first by the current filter (4) and (5). Then, the filtered current signal $\tilde{i}$ can be employed as the current feedback; the levitation gap signal is processed with the proposed tracking differentiator, one output is employed as the filtered gap feedback $\tilde{\delta }$, the other output of the tracking differentiator is employed as the differential feedback $\dot{\tilde{\delta }}$, which is used for suppression of the train-track coupled vibration; and, to diminish the static error, gap error is integrated as an additional feedback $\int (\delta -z_{ref})dt$. Then, the levitation control law can be concluded as :

(9) $$u_{c}=K_{z}\tilde{\delta }+K_{v}\int \tilde{a}+K_{c}\tilde{i}+K_{b}\dot{\tilde{\delta }}+K_{i}\int \left(\delta -z_{ref}\right),$$

where $K_{z}$, $K_{v}$, $K_{c}$, $K_{b}$, and $K_{i}$ are, respectively, the corresponding coefficients.

## 3. Nonlinear Second Order Discrete Tracking Differentiator Based on Boundary Characteristics

In this part, the core of this signal processing architecture, the tracking differentiator, is introduced in detail.

### 3.1. Preliminaries of the Discrete Tracking Differentiator

A typical form of the discrete tracking differentiator is [21,25,26,32]:

(10) $$\left\{\begin{array}{c} x_{1}\left(k+1\right)=x_{1}\left(k\right)+hx_{2}\left(k\right),\\ x_{2}\left(k+1\right)=x_{2}\left(k\right)+hf_{td}\left(x_{1}\left(k\right)-v_{in}\left(k\right),x_{2}\left(k\right),r,c_{0}h\right), \end{array}\right.$$

in which $x_{1}\left(k\right)$ and $x_{2}\left(k\right)$ are the state variables describing the tracking differentiator, $v_{in}\left(k\right)$ is the input signal of the tracking differentiator (usually the polluted signal), *h* is the discrete sampling time, $f_{td}$ is the control comprehensive function, and *r* and $c_{0}$ are, respectively, the rapid coefficient and filtering coefficient. Under the condition that the values of the parameter *r* and $c_{0}$ are chosen properly and comprehensive function $f_{td}$ works well, $x_{1}\to v_{in}$ and $x_{2}\to {\dot{v}}_{in}$ can be realized.

### 3.2. Procedure to Calculate the Control Comprehensive Function

According to optimal control theory for the linear time-invariant (LTI) system, for a given continuous second order cascaded integral system:

(11) $$\left\{\begin{array}{c} {\dot{x}}_{1}\left(t\right)=x_{2}\left(t\right),\\ {\dot{x}}_{2}\left(t\right)=f_{td}\left(t\right),\left|f_{td}\left(t\right)\right|\leq r, \end{array}\right.$$

with respect to cost index:

(12) $$J\left[f_{td}\left(t\right)\right]=\int_{0}^{\tau }1\;dt=\tau ,$$

the fastest optimal control strategy is [33,34]:

(13) $$f_{td}\left(t\right)=-r\cdot sign\left(x_{1}\left(t\right)+\frac{x_{2}\left(t\right)\left|x_{2}\left(t\right)\right|}{2r}\right),$$

and any state point $P(x_{1}\left(t\right),x_{2}\left(t\right))$ on the phase plane needs at most the changing sign once from $f_{td}\left(t\right)=r$ (or $f_{td}\left(t\right)=-r$ ) to $f_{td}\left(t\right)=-r$ (or $f_{td}\left(t\right)=r$). The sign changing takes place as soon as the point reaches the boundary line:

(14) $$x_{1}\left(t\right)+\frac{x_{2}\left(t\right)\left|x_{2}\left(t\right)\right|}{2r}=0.$$

When it comes to a discrete system, most of the time, the state point cannot change sign exactly when the point arrives at the boundary line since there is a one-step sampling (or “delay”). Under this condition, a chattering in which the state point moves back and forth the boundary line will happen, and this chattering will deteriorate the working performance of the controller. Thus, some special measures should be taken around the boundary line.

There are two boundaries in the phase plane, one is the switching line constituted by those initial points that converge to origin if control values are always $f_{td}=r$ or $f_{td}=-r$. This characteristic line can be defined as $\Gamma_{A}$:

(15) $$x_{1}\left(t\right)+\frac{x_{2}\left(t\right)\left|x_{2}\left(t\right)\right|}{2r}+\frac{1}{2}hx_{2}\left(t\right)=0.$$

The other characteristic line is constituted by those initial points that converge to the origin when control value adopts $f_{td}=r$ first and then $f_{td}=-r$ always or the control value adopts $f_{td}=-r$ first and then $f_{td}=r$ always. This characteristic line can be defined as $\Gamma_{B}$:

(16) $$x_{1}\left(t\right)-s\frac{x_{2}^{2}\left(t\right)}{2r}+\frac{5}{2}hx_{2}\left(t\right)-sh^{2}r=0,s=sign\left(x_{1}\left(t\right)+hx_{2}\left(t\right)\right).$$

The state points that are able to arrive at the origin within two steps are girdled by two pairs of parallel lines, and this region can be defined as reachable zone $\Omega_{r}$:

(17) $$\Omega_{r}=\{\left(x_{1}\left(t\right),x_{2}\left(t\right)\right)\left|\right|x_{1}\left(t\right)+2hx_{2}\left(t\right)|\leq h^{2}r,|x_{1}\left(t\right)+hx_{2}\left(t\right)\left|\leq h^{2}r\right\}.$$

**Figure 4:**
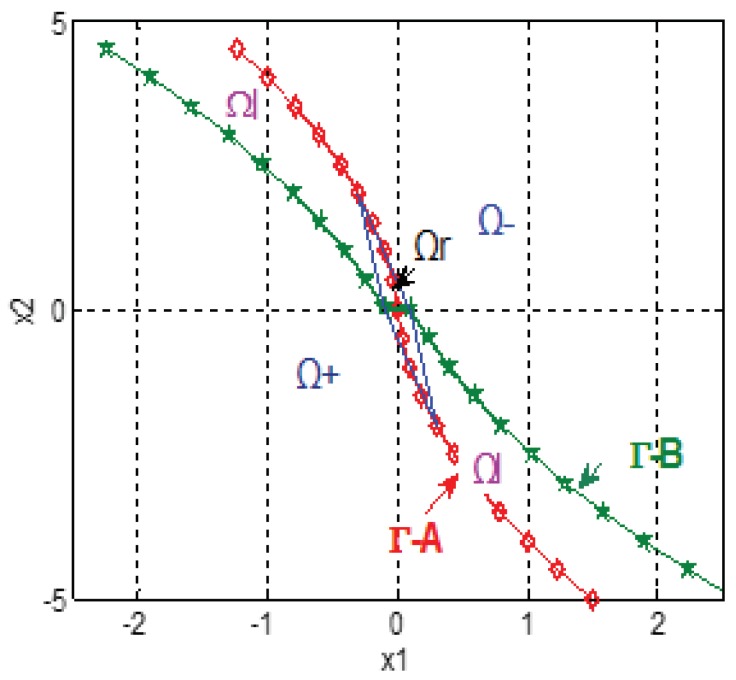
The division of phase plane.

Define the zone between $\Gamma_{A}$ and $\Gamma_{B}$ except $\Omega_{r}$ as linear zone $\Omega_{l}$. The other two zones are defined as negative zone $\Omega_{-}$: the zone on the right upward region, and positive zone $\Omega_{+}$: the zone on the left downward region. Thus, it can be seen in Figure 4 that the phase plane can be divided into four parts: $\Omega_{l}$, $\Omega_{r}$, $\Omega_{+}$ and $\Omega_{-}$.

It is obvious that, in region $\Omega_{-}$, $f_{td}=-r$; in the region $\Omega_{+}$, $f_{td}=r$. The difference for control strategy between continuous system and discrete system exists when the state point belongs to the linear region $\Omega_{l}$ and the reachable region $\Omega_{r}$. When state P(k) is in the linear region $\Omega_{l}$, to avoid the chattering, a linearly changing comprehensive function $f_{td}$ is chosen to make the next state P(k+1) moves along the switching line towards the origin steadily. Make a horizontal line through point P(k) on the phase plane, thus this horizontal line has two cross points with $\Gamma_{A}$ and $\Gamma_{B}$. Then, define the cross points as *A*, *B*, and their *x*-axis values are defined as $X_{A}$, $X_{B}$; then, a linearly changing control variable:

(18) $$f_{td}\left(t\right)=-r\frac{\left(X_{B}+X_{A}-2|x_{1}\left(t\right)|\right)}{X_{B}+X_{A}}sign\left(x_{2}\left(t\right)\right)$$

can be adopted to make the next state P(k+1) and all other following states fall within the linear region $\Omega_{l}$, and converge to the origin along the boundary $\Gamma_{A}$. When the state is within the reachable region $\Omega_{r}$, the control strategy can be derived from the system equation as:

(19) $$f_{td}\left(t\right)=-\frac{1}{h^{2}}\left(x_{1}\left(t\right)+2hx_{2}\left(t\right)\right).$$

**Figure 5:**
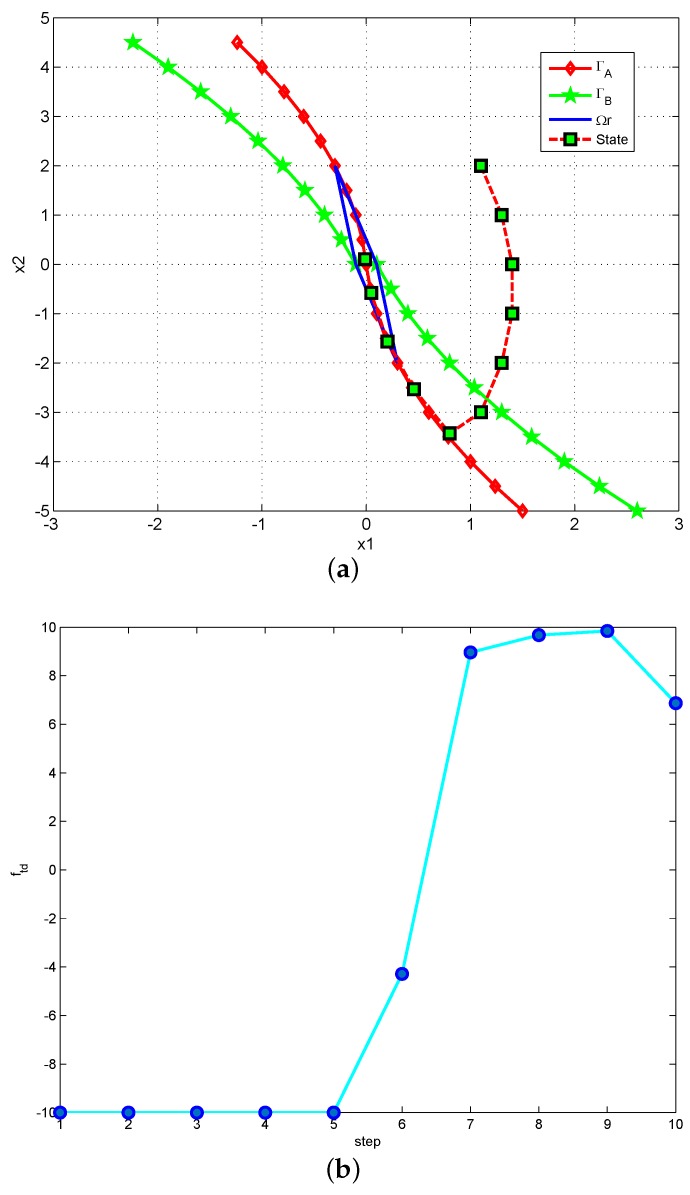
Convergence of a state point on the phase plane. (**a**) locus of the state point; (**b**) value of $f_{td}$.

In conclusion, the basic strategy of this nonlinear tracking differentiator is: given a point $P(x_{1},x_{2})$ in the phase plane, the value of control comprehensive function $f_{td}\left(x_{1},x_{2}\right)$ is determined so that the state point can converge to the origin as soon as possible. The strategy of calculating control comprehensive function $f_{td}$ is: make a horizontal line through point $P(x_{1},x_{2})$ on the phase plane, get the value of $X_{A}$ and $X_{B}$, then obtain the value of $f_{td}$ according to which zone $P(x_{1},x_{2})$ belongs to. As an instance, the locus for an initial point $P\left(x_{1},x_{2}\right)=\left(1.1,2.0\right)$ in phase plane can be seen in Figure 5a; here, the parameters are r=10, h=0.1, and $c_{0}=1$. In this figure, point $P(x_{1},x_{2})$ moves from zone $\Omega_{-}$ to the linear zone $\Omega_{l}$, entering reachable zone $\Omega_{r}$ along the switching line $\Gamma_{A}$, and eventually converging to the origin in 10 steps, with no fluttering during this whole process. The locus of the state point is so close to the optimal convergence locus owing to the proper choosing of control comprehensive function $f_{td}\left(x_{1},x_{2}\right)$, which can be seen in Figure 5b.

### 3.3. Influence of the Parameters

**Figure 6:**
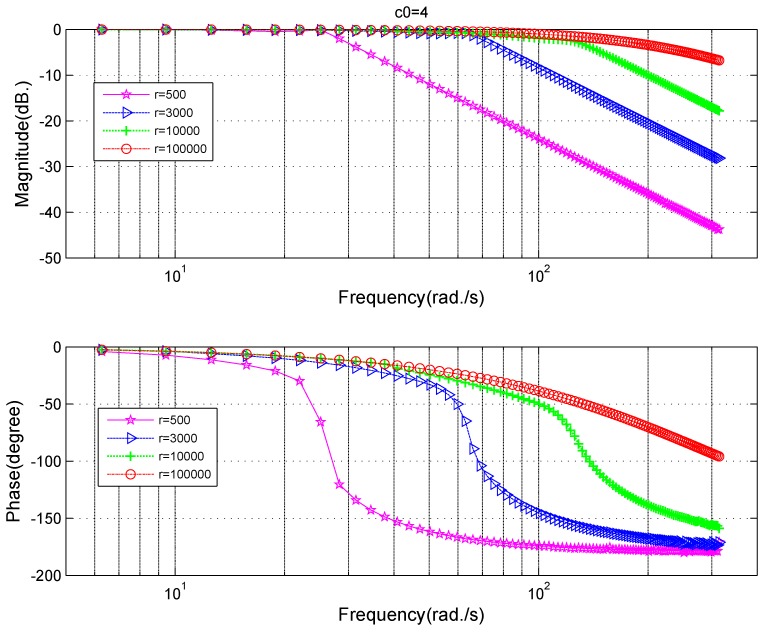
Frequency characteristics of the tracking differentiator at different fast coefficients *r* for filtering purposes.

**Figure 7:**
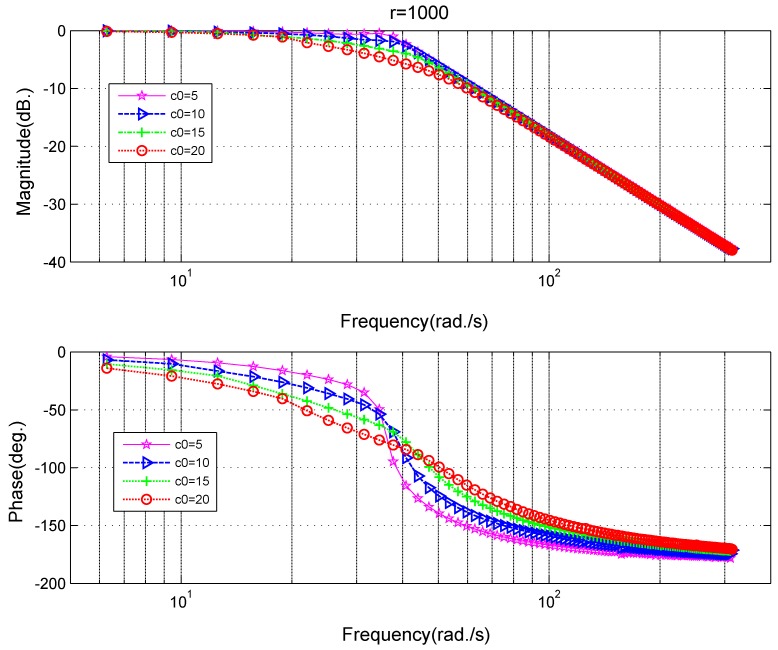
Frequency characteristics of the tracking differentiator at different filtering coefficient $c_{0}$ for filtering purposes.

The fast parameter *r* and filtering parameter $c_{0}$ are strongly related to the performance of the tracking-differentiator. In order to select proper parameters and make adjustments if it is needed, influences of different parameters on TD’s frequency characteristics are indispensable. A frequency scanning method can be employed to obtain these relationships. The frequency characteristics at different values of the parameter *r* can be seen in Figure 6. At the low-frequency section (frequency < 40 Hz), the amplitude gain is close to 1, the phase lag is small. At high-frequency section (frequency > 100 Hz), the amplitude gain decreases and phase lag increases, which makes it able to suppress high-frequency noise. The crossover frequency for amplitude gain increases and the phase lag decreases as *r* increases, which means the increase of *r* strengthens the tracking property while weakens the filtering property. The frequency characteristics at different values of the parameter $c_{0}$ can be seen in Figure 7. As $c_{0}$ increases, the amplitude gain decreases gradually, which means that the filtering ability increases and correspondingly the phase lag increases with $c_{0}$ at the same time. When $c_{0}=15$, the phase lag is pretty large, which is not a good thing for the system’s stability [3].

Based on the filtering frequency characteristics on different fast parameter *r* in Figure 6, on different filtering parameter $c_{0}$ in Figure 7, a trade-off between the phase lag and restraining effects for the noise can be made comprehensively. Supposing the filtering parameter is chosen as $c_{0}=10$, and the fast parameter is chosen as $r=6\pi \times 10^{6}$, when the frequency of the input sinusoidal signal is 50 Hz, the amplitude gain of the tracking differentiator is −0.048 dB, the ratio between the amplitude of input and output signal is 1:0.9945, the phase lag is 8.52 degree, and both the amplitude attenuation and the phase lag are tolerable.

**Figure 8:**
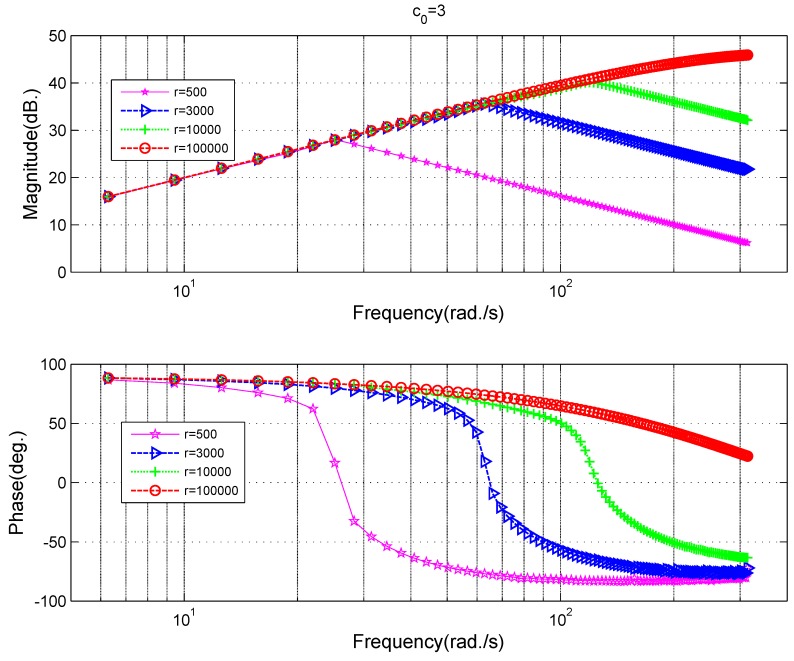
Frequency characteristics of the tracking differentiator at different fast coefficient *r* for differential purposes.

**Figure 9:**
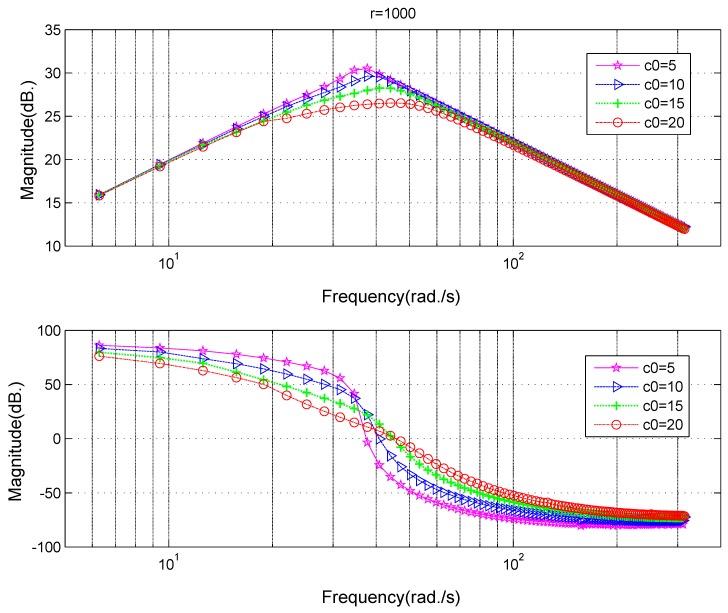
Frequency characteristics of the tracking differentiator at different filtering coefficient $c_{0}$ for differential purpose.

The influences of different parameters on the differential frequency characteristics of TD can be acquired in the same way. Figure 8 is the case for different values of parameter *r* and Figure 9 is the case for different values of parameter $c_{0}$. The explanation of these differential results are similar to those for the filtering case in Figure 6 and Figure 7, so it will not be made in detail. The key point is that there is a turning point for the amplitude–frequency relationship in Figure 8 and Figure 9,which means that the high frequency noise will not be amplified by the differential of TD compared with the classic differentiator (8).

**Remark** **1.** *For the differential, the turning point increases with the parameter r, which means that high frequency input signal needs a large parameter r. A large parameter $c_{0}$ will increase the ability of noise suppression, while, at the same time, affect the phase lag, so the value of $c_{0}$ should not be chosen too large.*

## 4. Simulation and Experiment Analysis

### 4.1. Simulation of TD Based Signal Processing Architecture in MATLAB-Simulink

**Figure 10:**
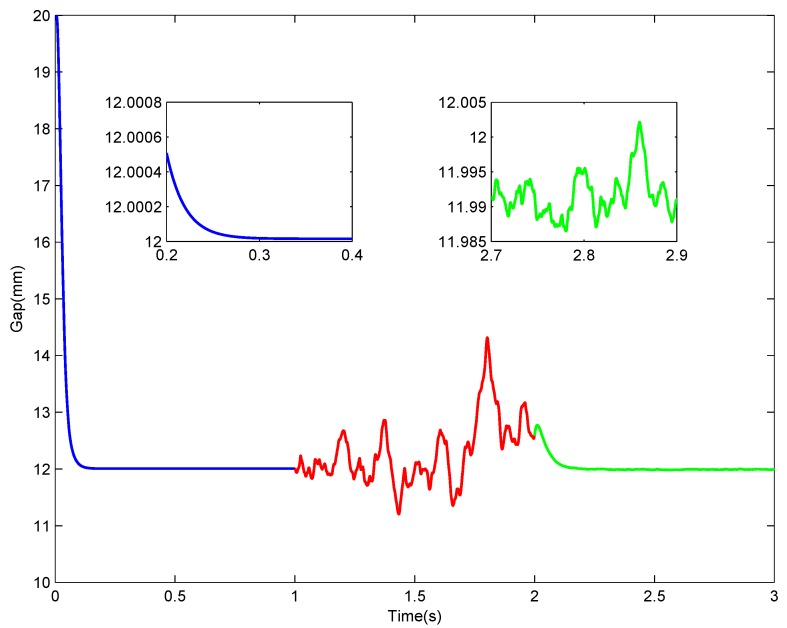
Levitation gap when the gap sensor signal is polluted by noise.

In this part, a maglev system with TD based signal processing architecture shown in Figure 3 is simulated in MATLAB-Simulink under control law (9). To highlight major issues and to save the space, only noise disturbance in gap sensor signal is considered in this part. The procedure of this simulation is: (1) it is an ideal condition with no sensor noise in the beginning 1 s, under the predesigned control law, the levitation gap will change from an initial value of 20 mm to a desired target value of 12 mm; (2) after the system stabilizes at the equilibrium point, white noise with mean value 0 and magnitude 0.25 mm is added into the gap signal from t=1 s to simulate the disturbed gap signal; and (3) signal processing architecture proposed in this paper is then adopted to process the noised gap signal from t=2 s. After this simulation, the levitation gap value during this whole process is recorded and displayed in Figure 10. The levitation gap at different time sections is distinguished with different colors, and partial zooms are made in the first and last section, respectively. After the simulation begins, the levitation gap diminishes quickly and smoothly from the initial value to the target value within 0.4 s, with no overshoot and no static error. When noise is added into the gap sensor without processing, the levitation gap begins to fluctuate dramatically and the deviation is about 3 mm the maximum. Then, after the signal processing method is employed, the fluctuation is suppressed within 0.015 mm, which can be seen in the partial zoom window. From a statistical point of view, the mean value of gap and its variance when noise is added is 12.2875 and 0.32, respectively, while when the filter is employed, it is 12.0384 and 0.0216, respectively. This simulation presents a demonstration for the influence of the gap sensor noise and the effective performance of the proposed signal processing architecture.

### 4.2. VHDL Implementation and MATLAB-Modelsim Simulation for TD

FPGA is widely used in signal processing with the advantages of high speed and mass processing [35,36]. In the maglev system, an FPGA processor is employed in charge of signal processing tasks. Therefore, to make this TD able to function in a real maglev levitation system, a VHDL implementation has to be made to make it work in an FPGA processor. In addition, this TD’s performance can be tested in a simulation first. Usually, a VHDL program is simulated in simulation software Modelsim (Modelsim 14.1, Mentor Graphics, Wilsonville, OR, USA). However, Modelsim is not powerful enough in generating desired input signal, while MATLAB has the advantages of flexible input signal configuration and powerful input–output data process ability. In this section, the new MATLAB-Modelsim simulation is introduced in detail and the results are given. A MATLAB-Modelsim simulation process can be completed in five steps:

(1) Generate the desired input signal in a MATLAB file and store the signal data into a text document. In this process, signals with almost any properties can be generated easily.
(2) Import of the input signal. In this step, Modelsim reads the text document generated in step (1), and import the stored data as the input data for the tracking differentiator.
(3) Operation of tracking differentiator. This is the core step of this entire simulation.
(4) Storage of the output data. All data are stored in a text file.
(5) Reading of the output data and make some necessary signal processing via MATLAB’s signal processing tools.

**Figure 11:**
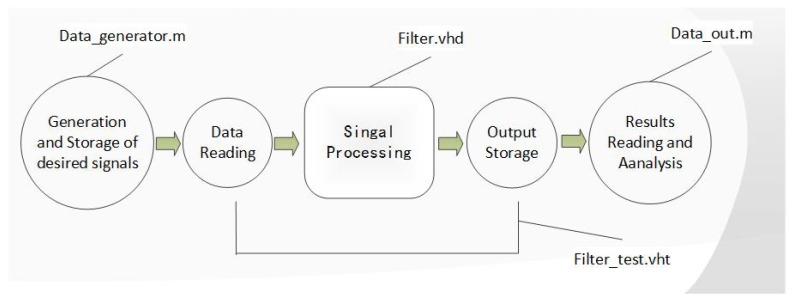
MATLAB-Modelsim Simulation procedure.

This procedure and corresponding files used can be seen in Figure 11. All simulation files are available in supplementary materials attached.

Given a gap signal *w*, which comprises of a 12 mm constant component, a 50 Hz, 1 mm sinusoidal component, and 0.1 mm white noise. Through a digitization process in (20), this gap signal is converted into a 12-bit digital signal:

(20) $$w_{n}=round\left(w/20\times \left(2^{12}-1\right)\right).$$

**Figure 12:**
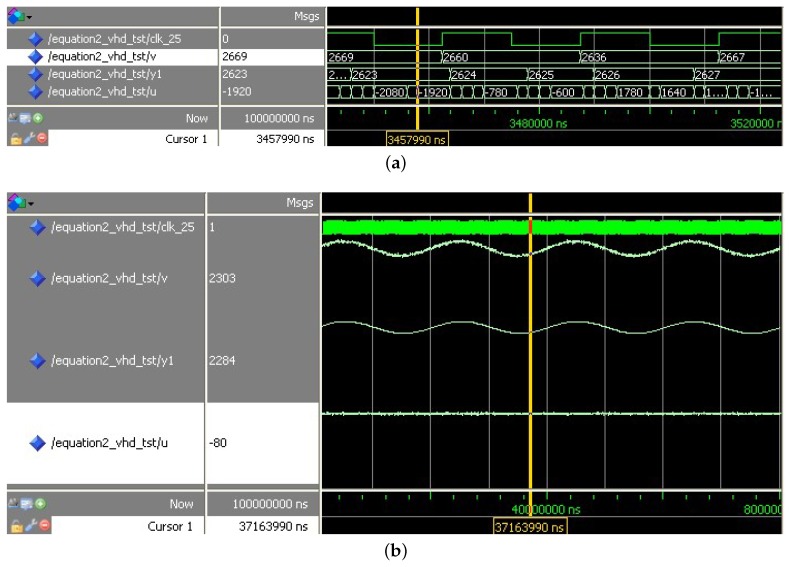
Simulation result in Modelsim. (**a**) a binary waveform display of simulation result in Modelsim; (**b**) an analog waveform display of simulation results in Modelsim.

Through a simulation in Modelsim, the simulation result is shown in Figure 12a. This result consists of three columns: signal name, value and the wave. In the column “signal name”, clk_25 is the sampling clock, *v* is the input signal, y1 is the filtered output, and *u* is the control comprehensive function. If a cursor is placed on the wave, the corresponding value can be seen in the middle column. In Modelsim, waveforms can be displayed in different formats: binary, decimal or hex. Especially, the result can be displayed in analog waveforms, like that in Figure 12b. Comparing the input signal with the output signal , the output signal can track the input signal and the noise in the output signal is suppressed, which in turn indicates that the VHDL program is effective. A simulation for differential of TD is more or less the same so the simulation result for the differential is omitted here.

**Figure 13:**
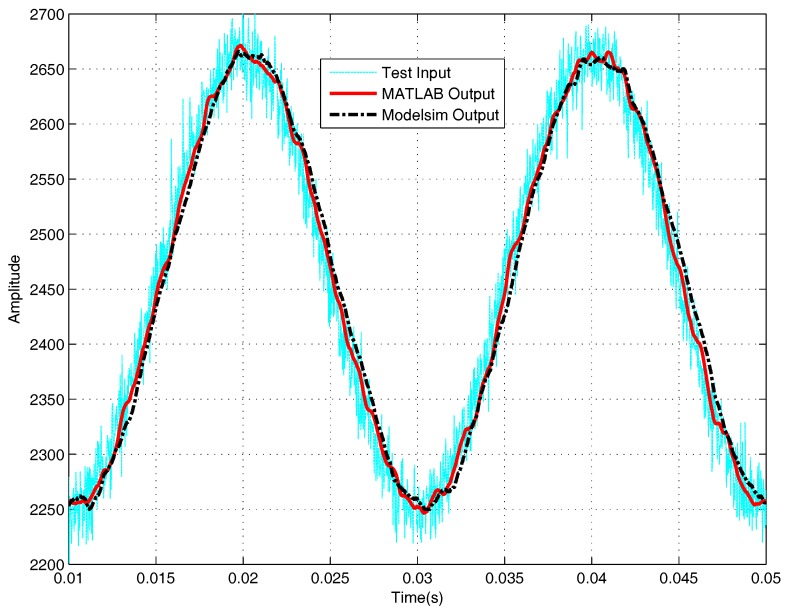
Comparative simulation performances for filtering in MATLAB and Modelsim.

**Figure 14:**
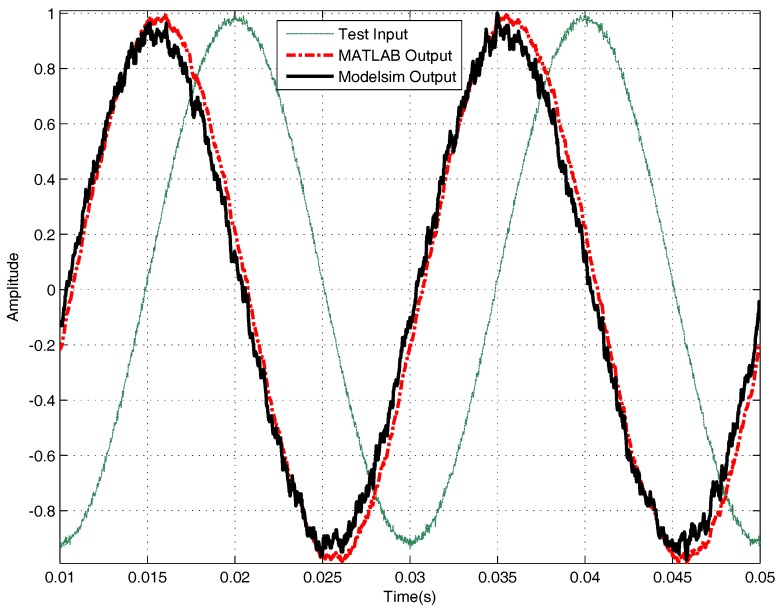
Comparative simulation performances for differential in MATLAB and Modelsim.

To get a more precise analysis of the filter’s performance, observing the waveform in Modelsim is neither convenient nor sufficient. Therefore, processing all these input and output data in MATLAB is a better way. First, computation in MATLAB has a more precise result, which can be treated as a standard to judge whether the result obtained from the VHDL program is accurate or not. Second, MATLAB has a more powerful signal processing ability, not only displaying the signal in the time domain but also displaying the signal in frequency domain. Figure 13 and Figure 14 are comparisons of the computation results in VHDL and the computation results in MATLAB. For filtering, the average error between MATLAB result and VHDL result is 0.509, the ratio of this error to the mean value of the output signal is 0.02%; for the differential, the average error between MATLAB result and VHDL result is 2.316, the ratio of this error to the mean absolute value of the output signal is 0.21%. The two results are very close; both the red signals obtained in MATLAB and the black signals obtained in Modelsim are effective processed results. Assuming the results in MATLAB are accurate, the simulation results indicate that the computation results in VHDL are precise as well.

**Remark** **2.** *The differential results in Figure 14 are normalized results; here, only the “shape” of the waves are considered, and the normalization is convenient for comparison.*

### 4.3. Experiment Analysis of the Proposed Signal Processing Architecture

**Figure 15:**
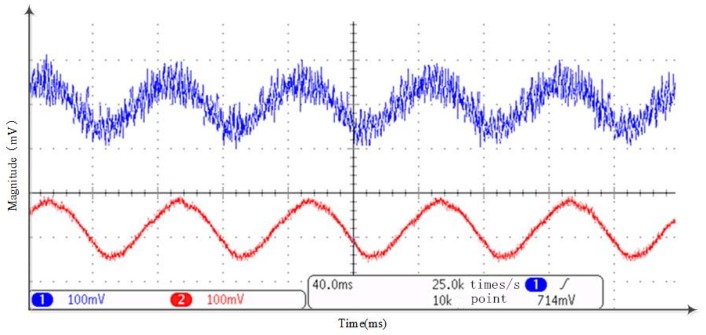
Tracking differentiator (TD) test for filtering at an onboard test bench.

**Figure 16:**
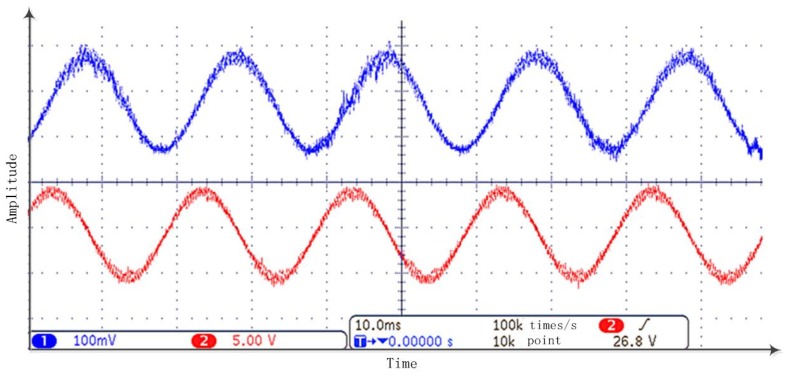
TD test for differentiating at an onboard test bench.

After the MATLAB-Modelsim simulation, an independent experiment is made to check out this TD’s performance in an FPGA processor. The idea is generating the desired test signal, processing it in an FPGA processor and making a comparison between the input and the output signals in an oscilloscope. Given a 50 Hz sinuous test input signal, the filtering performance is shown in Figure 15 while the differential performance is shown in Figure 16. In both figures, the blue signal in channel 1 is the input signal and red signal in channel 2 is the processed signal. It can be concluded that both filtering and differential results are satisfactory.

**Figure 17:**
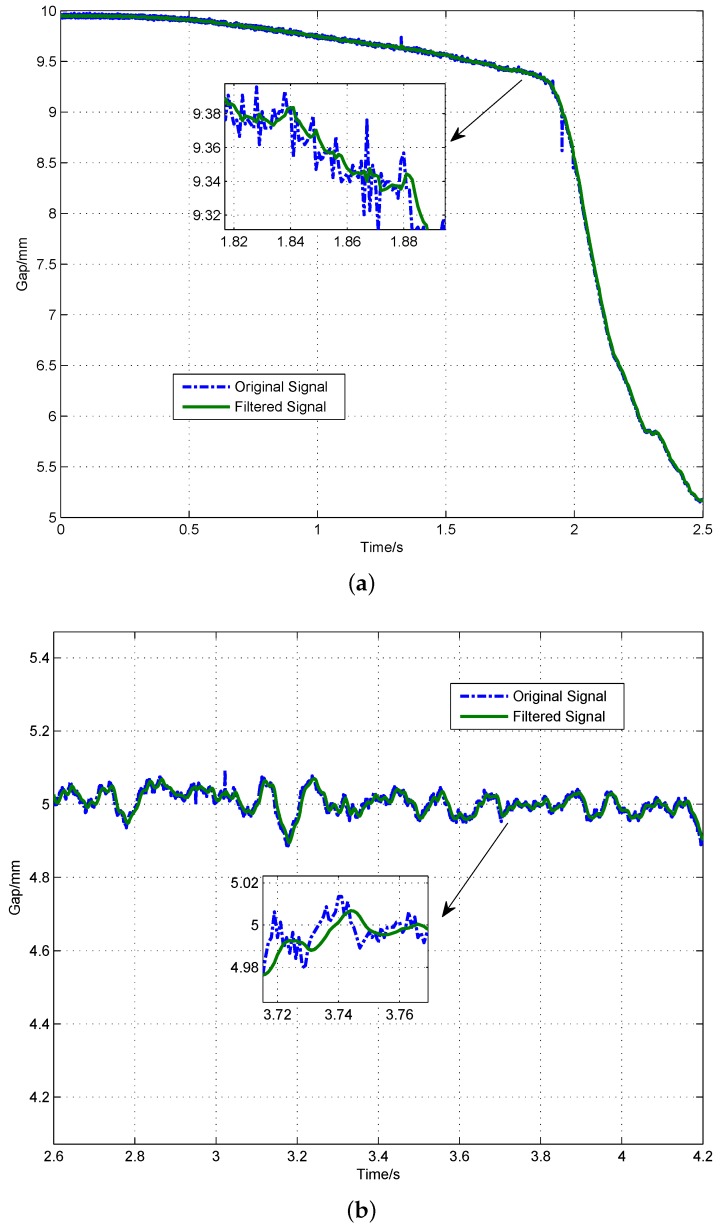

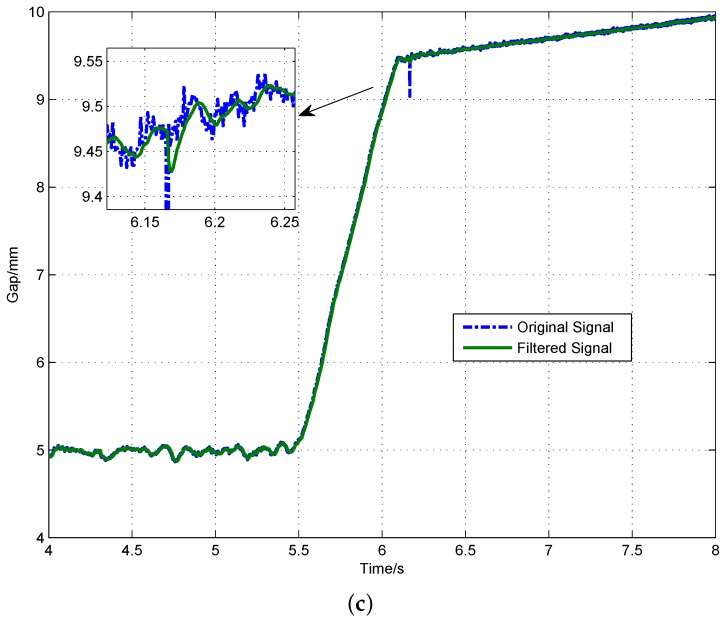
Original gap signal and filtered signal. (**a**) rising process; (**b**) levitation process; and (**c**) falling process.

**Figure 18:**
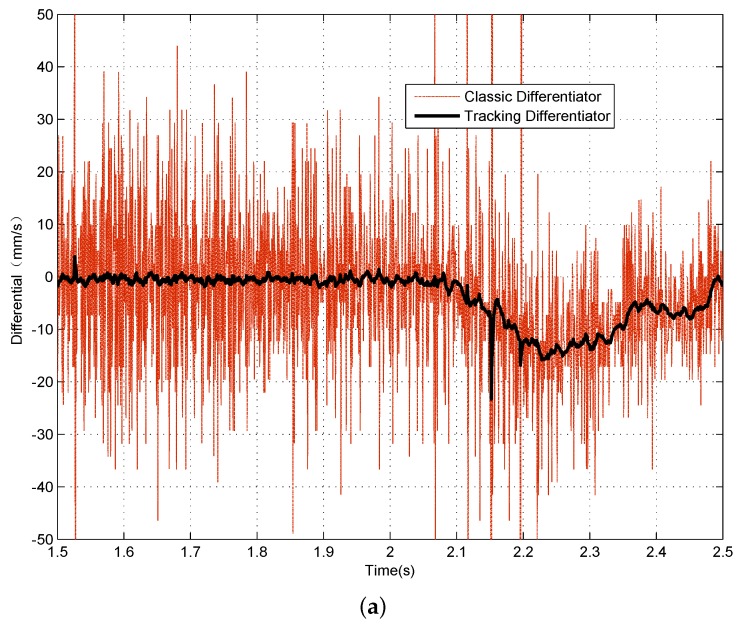

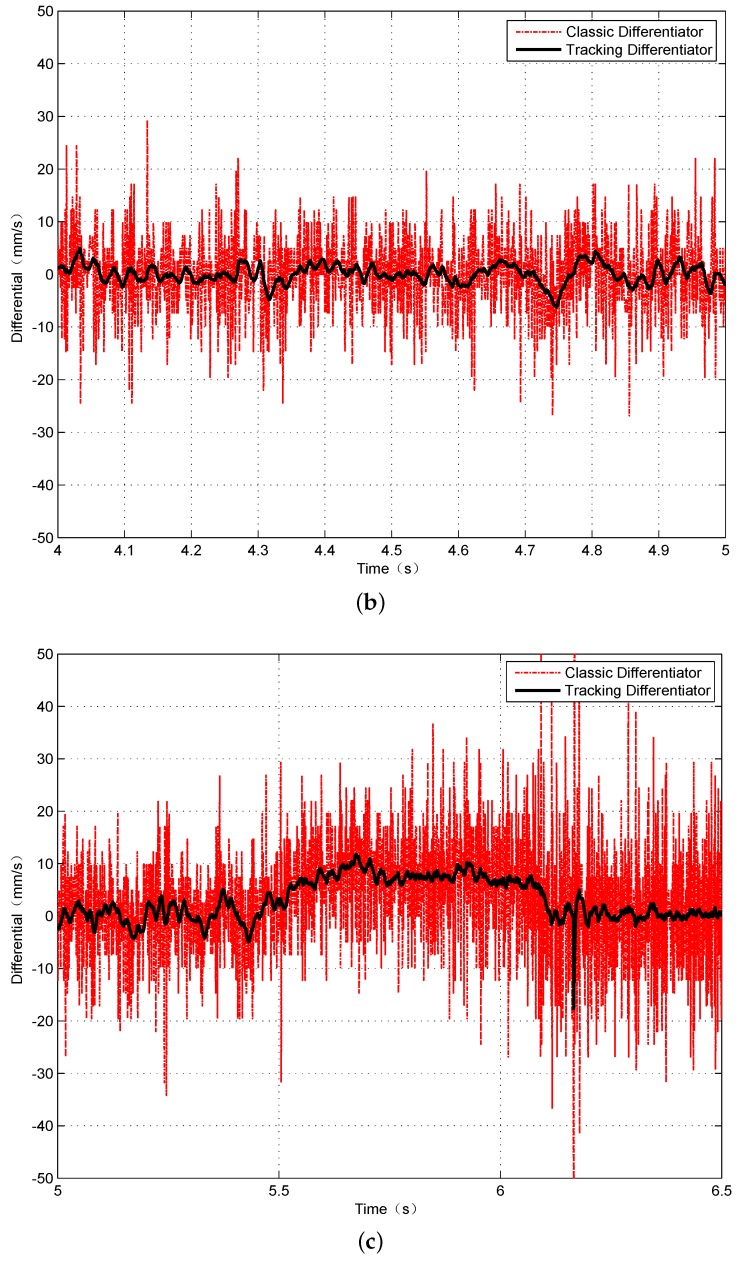
Gap signal differential results. (**a**) rising process; (**b**) levitation process; (**c**) falling process.

The final step is to verify the effectiveness of the signal processing algorithm in a real maglev system. For this purpose, an experiment on a maglev test bench is performed. The designed signal processing architecture is applied on the sensors’ signals, and control output value is computed taking the processed signals as the feedback. The experiment procedure is making this maglev vehicle levitate from initial gap $x_{0}=10$ mm to a desired set value of $x_{0}=5$ mm and then fall off from the target position to its initial position. The performance of the signal processing algorithm is shown in Figure 17 for the filtering and in Figure 18 for the differential. The results show that this signal processing architecture can make the maglev train levitate steadily, and, in steady state, the sensor noise is filtered and the track-train coupled vibration is effectively suppressed.

**Figure 19:**
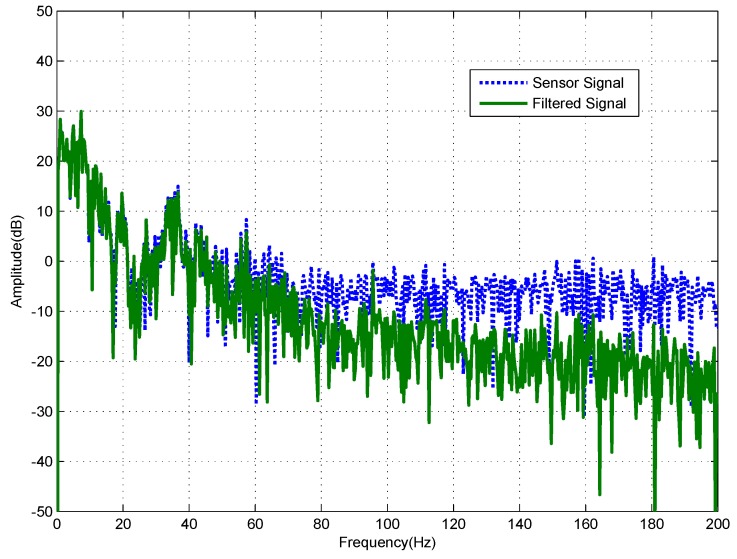
Frequency spectrum of the input and output signals.

In addition, the filter’s performance for filtering in the frequency domain can be seen in Figure 19, which shows that the filter can maintain the signal’s less than 50 Hz low-frequency components while suppressing the high frequency noise disturbance effectively.

## 5. Conclusions

A tracking differentiator based signal processing architecture for a maglev train levitation system is proposed and evaluated in this paper for its performance and practicality. Design and tuning issues for the core of this signal processing architecture, the tracking differentiator, is studied in detail. The advantages of this nonlinear discrete time tracking differentiator are demonstrated in MATLAB-based simulations, VHDL-based implementation and maglev train signal processing. In particular, this new type of tracking differentiator can suppress the noise effectively with a relatively small phase lag and can acquire the differential for a given signal without amplifying the high frequency noise. The work of this paper is helpful for maglev train signal processing and for the usage of a tracking differentiator in a real system. Further research and applications can be done based on this work. 

## Supplementary Materials

The following are available online at http://www.mdpi.com/1424-8220/18/6/1697/s1.
